# Neural Mechanisms for Accepting and Rejecting Artificial Social Partners in the Uncanny Valley

**DOI:** 10.1523/JNEUROSCI.2956-18.2019

**Published:** 2019-08-14

**Authors:** Astrid M. Rosenthal-von der Pütten, Nicole C. Krämer, Stefan Maderwald, Matthias Brand, Fabian Grabenhorst

**Affiliations:** ^1^Social Psychology: Media and Communication, University Duisburg-Essen, 47048 Duisburg, Germany,; ^2^Individual and Technology, RWTH Aachen University, 52062 Aachen, Germany,; ^3^Erwin L. Hahn Institute for Magnetic Resonance Imaging, 45141 Essen, Germany,; ^4^General Psychology: Cognition and Center for Behavioral Addiction Research (CeBAR), University Duisburg-Essen, 47048 Duisburg, Germany, and; ^5^Department of Physiology, Development and Neuroscience, University of Cambridge, CB2 3DY Cambridge, United Kingdom

**Keywords:** emotion, mentalizing, prefrontal cortex, reward, social

## Abstract

Artificial agents are becoming prevalent across human life domains. However, the neural mechanisms underlying human responses to these new, artificial social partners remain unclear. The uncanny valley (UV) hypothesis predicts that humans prefer anthropomorphic agents but reject them if they become too humanlike—the so-called UV reaction. Using fMRI, we investigated neural activity when subjects evaluated artificial agents and made decisions about them. Across two experimental tasks, the ventromedial prefrontal cortex (VMPFC) encoded an explicit representation of subjects' UV reactions. Specifically, VMPFC signaled the subjective likability of artificial agents as a nonlinear function of humanlikeness, with selective low likability for highly humanlike agents. In exploratory across-subject analyses, these effects explained individual differences in psychophysical evaluations and preference choices. Functionally connected areas encoded critical inputs for these signals: the temporoparietal junction encoded a linear humanlikeness continuum, whereas nonlinear representations of humanlikeness in dorsomedial prefrontal cortex (DMPFC) and fusiform gyrus emphasized a human–nonhuman distinction. Following principles of multisensory integration, multiplicative combination of these signals reconstructed VMPFC's valuation function. During decision making, separate signals in VMPFC and DMPFC encoded subjects' decision variable for choices involving humans or artificial agents, respectively. A distinct amygdala signal predicted rejection of artificial agents. Our data suggest that human reactions toward artificial agents are governed by a neural mechanism that generates a selective, nonlinear valuation in response to a specific feature combination (humanlikeness in nonhuman agents). Thus, a basic principle known from sensory coding—neural feature selectivity from linear–nonlinear transformation—may also underlie human responses to artificial social partners.

**SIGNIFICANCE STATEMENT** Would you trust a robot to make decisions for you? Autonomous artificial agents are increasingly entering our lives, but how the human brain responds to these new artificial social partners remains unclear. The uncanny valley (UV) hypothesis—an influential psychological framework—captures the observation that human responses to artificial agents are nonlinear: we like increasingly anthropomorphic artificial agents, but feel uncomfortable if they become too humanlike. Here we investigated neural activity when humans evaluated artificial agents and made personal decisions about them. Our findings suggest a novel neurobiological conceptualization of human responses toward artificial agents: the UV reaction—a selective dislike of highly humanlike agents—is based on nonlinear value-coding in ventromedial prefrontal cortex, a key component of the brain's reward system.

## Introduction

Would you trust a robot to make personal choices for you? Artificial agents capable of decision making are becoming more prevalent across human life domains (Broadbent, 2017). Such artificial (i.e., synthetic, not naturally occurring) agents can elicit positive emotions but they can also make humans uncomfortable and even induce repulsion, leading to rejection as social partners (Mori et al., 2012; Broadbent, 2017). Understanding human responses to artificial agents is important, not only for optimizing human-robot interaction, but it may also reveal previously unrecognized mechanisms governing human–human social interactions (MacDorman and Ishiguro, 2006; Vogeley and Bente, 2010).

The influence of physical appearance on human acceptance of artificial agents is conceptualized by the uncanny valley (UV) hypothesis (Mori, 1970; Mori et al., 2012). This hypothesis states that robots become more likable the more humanlike they appear. But, when robots become too humanlike, likability decreases and instead reverses into negative reactions of dislike, eeriness or uncanniness. This nonlinear valuation is the so-called UV reaction. Although its psychological basis is not fully understood (Rosenthal-von der Putten and Kramer, 2014; Rosenthal-von der Putten and Weiss, 2015; MacDorman and Chattopadhyay, 2016; Lischetzke et al., 2017), the UV hypothesis offers a framework for investigating human–robot interactions and associated neural mechanisms.

From a neural coding perspective, human responses to artificial agents suggest a transition from a linear representation of humanlikeness to a nonlinear representation of likability (i.e., selectively decreased likability for highly humanlike agents). In sensory systems, such linear–nonlinear transformations occur gradually as neurons acquire selectivity to specific feature combinations (Olshausen and Field, 2004). However, it is unexplored whether this coding principle also underlies responses to artificial agents.

Here, we used fMRI to investigate the neural mechanisms underlying reactions toward artificial agents and their role in decision making. We hypothesized involvement of neural systems supporting mentalizing and social perception, including temporoparietal junction (TPJ) and dorsomedial prefrontal cortex (DMPFC) (Saxe and Wexler, 2005; Amodio and Frith, 2006; Mitchell et al., 2006; Hampton et al., 2008; Behrens et al., 2009; Bhatt et al., 2012), as well as systems supporting valuation and decision making, including ventromedial prefrontal cortex (VMPFC) (Chib et al., 2009; Hare et al., 2010; Bartra et al., 2013; Neubert et al., 2015). We examined these areas' contributions to UV reactions by measuring neural activity in two paradigms—a psychophysical rating task and a choice task. We addressed the following questions.

First, it is unclear whether a subjective UV reaction is itself neurally represented. Neural signals related to anthropomorphism of artificial agents have previously been described (Krach et al., 2008); however, an explicit neural UV representation has remained elusive (Cheetham et al., 2011). Evidence for a neural UV representation would support the UV hypothesis as a principle governing human–robot interactions, with potential broader implications for human–human interactions.

Second, the UV hypothesis implies the existence of: (1) a system that derives humanlikeness from sensory cues and (2) a downstream system that integrates these signals to a nonlinear value function, encoding low likability specifically for humanlike artificial agents. Analogous nonlinear responses to specific feature-combinations occur in multisensory integration (de Araujo et al., 2003; Stein and Stanford, 2008). But whether a similar principle applies to social valuations—including UV reactions—is unknown.

Third, as previous imaging studies focused largely on perception of robots (Chaminade et al., 2007; Krach et al., 2008; Cheetham et al., 2011), the neural processes underlying decision making about artificial agents remain unclear. These processes likely converge in VMPFC and DMPFC, where mentalizing and decision networks intersect (Bartra et al., 2013; Wittmann et al., 2018). Recent studies showed that activity in these areas differs during social choices (Nicolle et al., 2012; Wittmann et al., 2016) and evaluations (Mitchell et al., 2006; Jenkins et al., 2008). Despite these advances, delineating functional distinctions between VMPFC and DMPFC remains difficult and approaches involving artificial agents, as taken here in the context of the UV hypothesis, may offer new insights.

## Materials and Methods

### 

#### 

##### Participants.

Twenty-six healthy volunteers (14 female, 12 male; aged between 18 and 35 years, M = 23.04, SD = 4.47) participated in this study, though the final sample consisted of 21 subjects (see below). The local ethical committee approved the study. Participants were recruited via general advertising on campus. Inclusion criterion was that participants had to be aged between 18 and 35 years. Exclusion criteria were the usual exclusion criteria due to technical and medical limitations (no implants; no large tattoos in the region of head, neck, shoulder and upper back; no claustrophobia; no current medication). None of the 26 participants suffered from neurological or psychiatric diseases as ensured by previous E-mail-based screening. Twenty-four participants were right-handed and two left-handed. Four data sets were excluded due to data loss or technical problems during scanning. Most of participants were students (*n* = 23) who received extra credit (hourly credit as a trial subject). Nonstudent participants (*n* = 3) were reimbursed with €40. Upon arrival participants were instructed and signed informed consent. Before starting the scanning procedure, participants trained how to operate the response device used in the scanner. They completed a series of rating tasks and a series of choice tasks. These rating and choice tasks involved different pictures as subsequently used in the scanner. Subsequently, participants were prepared for the scanner and then completed the six experimental sessions in the scanner. After the fMRI session, participants completed a questionnaire after which they were debriefed, reimbursed and thanked for their participation.

##### Design.

Participants underwent six experimental sessions of fMRI scanning of the main task and two additional shorter sessions for functional localizers (not analyzed for the present study). In each experimental session, participants were asked to perform rating and choice tasks, which were run in random permutation trial-by-trial. In total, participants performed 72 trials of the rating task and 108 trials of the choice task.

##### Stimuli.

The experiment used six stimulus categories: humans without physical impairments, humans with physical impairments, artificial (synthetic) humans, android robots, humanoid robots and mechanoid robots.

Pictures of humans with and without physical impairments were taken from picture databases (www.shutterstock.com; www.gettyimages.com, www.istockphoto.com, www.fotolia.de). Only pictures showing people in a standing (if possible for humans with physical impairments), frontal position without exaggerated postures or exaggerated facial expressions in front of a white background were considered for the pretest. Pictures with extreme colors (e.g., bright red) were excluded.

Pictures of artificial humans were created based on portraits of people who received extreme plastic surgery (Toledano, 2011). The pictures present the people in dramatic light and reduced coloring (resulting in a light-gray complexion). According to the orientation of the heads depicted in the Toledano portraits, pictures were taken of volunteers exposing the same head and body orientation, under similar light conditions. The pictures of the bodies were also reduced in coloring and matched to the portraits, resulting in full body images of humans who share some irritating features: reduced coloring which resulted in light-gray complexion, mismatches in the proportion of head and body, exaggerated facial features (due to plastic surgery). In total, nine synthetic humans (four female, five male) were evaluated.

For the category of the android robots, the pretest included a set of 10 android robots. Again, pictures showed the robots in a standing or sitting frontal position without exaggerated postures or exaggerated facial expressions.

For the humanoid and mechanoid robots, pictures were chosen from a study on robot appearances (Rosenthal-von der Putten and Kramer, 2014). In total, 10 mechanoid and eight humanoid robots were evaluated.

In the pretest, the humans and robots were evaluated with regard to eight items (likable, unpleasant, familiar, uncanny, intelligent, disgusting, humanlike, and attractive) rated on a six-point Likert scale ranging from “I fully agree” to “I do not agree at all”. To keep the test short, two sets of pictures were created which were evaluated by 77 participants (39 participants completed set one, and 38 completed set two). Mean values for each of the eight items and each of the stimulus pictures were calculated. With regard to the humans without physical impairments, those pictures of female and male healthy humans were selected that provided the best fit of high likability, humanlikeness, and attractiveness and rated low in terms of being uncanny, unpleasant and disgusting.

The resulting stimulus material consists of six pictures for each of the six categories. When necessary, and if possible, gender of the stimuli was balanced. However, due to the restricted original material it was not possible to balance for gender within the category of humans with impairments and synthetic humans. Both groups contained more pictures with male than with female people.

Thus, we wished to study evaluations for specific categories of stimuli that feature in the literature of the UV and that are distinguished from each other by their design features. These categories included: (1) the very un-humanlike mechanoid robots, which are typically not designed with the intention to mimic human appearance, (2) humanoid robots, which resemble humans in terms of basic body shape but without clear recognizable facial features, (3) android robots, which are explicitly designed to closely mimic human appearance including in facial features, (4) the newly introduced artificial humans, which were derived from real human faces (see our explanation above), and (5,6) humans with or without physical disabilities, which were recognizably human but varied in familiarity. Each of these categories was represented by several stimulus exemplars that were carefully selected based on pretests: In an online study (Rosenthal-von der Putten and Kramer, 2014), >40 robots were evaluated regarding UV measures. A cluster analysis showed that robots with similar patterns of received evaluations (e.g., on humanlikeness and likability) clustered also regarding design characteristics. Thus, our fMRI study was intended to study specific classes of stimuli based on theoretical and empirical considerations. This approach also closely follows the UV literature in which the UV effect is fit to mean data for stimulus categories (Burleigh and Schoenherr, 2015).

Visual stimulus presentation was controlled using the software PRESENTATION (Neurobehavioral Systems).

##### Rating task.

The rating trials started with the presentation of a stimulus for 4 s, followed by a blank screen for 3 s. Afterward, participants rated the stimulus with regard to its likability, familiarity and humanlikeness on three separate visual analog scales, each presented for 3 s. The scales ranged from 1 (not at all likable/familiar/humanlike) to 5 (very likable/familiar/humanlike). The rating scales were followed by a variable intertrial interval (ITI) with jittered duration of 2–6 s. An instruction was presented during the ITI (“rate” or “decide”) to inform participants about whether the next trial would be a rating or choice trial. Each specific picture (i.e., stimulus) was presented twice, resulting in 72 rating trials in total (6 stimulus categories × 6 pictures × 2 repetitions).

##### Choice task.

The choice trials started with the presentation of the first stimulus for 4 s, followed by a blank screen of 3 s. Then the second stimulus was shown for 4 s, followed by a blank screen of 4 s. Subjects were then prompted to report their choice by showing the options “first” and “second” on the monitor (within 2 s). Subsequently, participants rated their confidence level with regard to the previously reported decision on a separate visual analog scale presented for 3 s. The scale ranged from 1 (not at all confident) to 5 (very confident).

Subjects were instructed that they have to choose between two pictures against the background of the following scenario: “Before this study we asked all humans and robots to choose one item among four items which will be given to the volunteers as gratification for participation in this study. The four items were a movie theater voucher, a package of dishwasher tabs, a bottle of sparkling wine, and a package of quality toilet bowl deodorizer blocks. Every person and robot made a choice. You will see pictures of all these persons and robots, but will not receive information on who decided in favor for what item. During the choice task trial you will see two pictures each showing a person or a robot (in the possible combinations person-person, robot-person, and robot-robot). You shall decide in favor of the picture showing the robot or the person from whom you prefer to receive the previously chosen present. Since the time for decision making and reporting your decision is very short, please make a “gut decision”. There will be easy and hard decisions. Thus, please indicate your level of confidence in the decision on a subsequent rating scale from 1 (not at all confident) to 5 (very confident).”

All subjects received the same gift at the end of the session (a cinema voucher). They were instructed about the possible gifts they could receive with the information that the robots and humans would have decided for a gift before the experiment. The instruction contained pictures of four possible gifts, and samples of these gifts were placed on a shelf in the corner of the training room. Although the experimenter did not refer to these samples, they were visible to the subjects. Subjects typically asked questions about the choice task and the gifts; for instance, they confirmed with the experimenter that everybody (humans and robots) would have made a gift choice. Overall, subjects' behavior during instructions and debriefing did not indicate that they did not believe the cover story. (For example, nobody explicitly stated that they did not believe the cover story.)

The choice trials were designed as follows. In total, nine “choice contrasts” between the six categories of stimuli (humans without physical impairments, humans with physical impairments, artificial humans, android robots, humanoid robots, mechanoid robots) were implemented. These choice contrasts are shown on the *x*-axis of Figure 5*B*. The stimulus category of humans without physical impairments was used as a reference to be compared with all other stimulus categories in five of these choice contrasts; the android stimulus category was used as a reference to be compared with all other stimulus categories (except humans without physical impairments) in the remaining four choice contrasts. Within each choice contrast, there were 12 individual choice trials that contrasted specific stimulus exemplars from each category (e.g., a specific human stimulus vs a specific artificial human stimulus). Each of these 12 individual choice trials, comparing a pair of specific stimuli, occurred only once in the experiment. With respect to the order of presentation on each trial, these 12 choice trials were balanced: six comparisons started with a picture from one stimulus category and six started with pictures of the other category. This design resulted in a total of 108 choice trials (12 choice trials contrasting specific stimuli × nine choice contrasts).

##### Behavioral data analysis.

**Figure 1.**
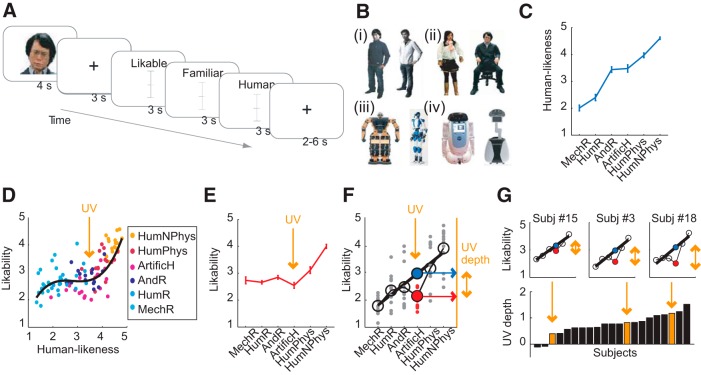
Behavioral UV reactions. ***A***, Rating task. Subjects evaluated UV-relevant dimensions of humans and artificial agents. ***B***, Example stimuli. (***Bi***) Artificial humans; (***Bii***) androids; (***Biii***) humanoid robots; (***Biv***) mechanoid robots. Human examples are not shown. ***C***, Humanlikeness ratings for stimulus categories (MechR: mechanoid robots; HumR: humanoid robots; AndR: android robots; ArtificH: artificial humans; HumPhys: humans with physical impairments; HumNPhys: humans without physical impairments). Relatively lower ratings were found for HumNPhys compared with HumPhys due to lower familiarity (see text). ***D***–***G***, UV effect in likability. ***D***, Likability ratings for all stimuli. Black line: third-order polynomial fit (*R*^2^ = 0.573). ***E***, Likability for artificial humans showed the UV-characteristic drop, indicating deviation from linear relationship between humanlikeness and likability. ***F***, Quantifying UV depth. UV definition in single subject's likability ratings. Black line: linear regression-fit of likability to humanlikeness continuum from all stimuli (gray data) except UV-relevant artificial humans (red data). Blue point: predicted likability for artificial humans from linear fit; red point: observed likability. UV depth defined as difference between predicted and observed likability. ***G***, UV depths across subjects. The procedure in ***F*** defined UV depths, illustrated for three individual subjects (top) and all subjects (bottom; *p* = 3.47 × 10^−8^, one-sample *t* test). UV effects were not found in several controls (e.g., humanoid robots; *p* > 0.15, one-sample *t* test). Error bars indicate SEM.

To analyze the rating data, we performed one-way repeated-measures ANOVA with stimulus category (shown on *x*-axis of Fig. 1*C*) as factor. Separate ANOVAs were performed for ratings of likability, humanlikeness and familiarity. We tested relationships between these rating variables using Pearson correlation and linear regression.

We focused on the artificial human stimuli to investigate the UV effect for the following reasons. These stimuli were conceptually very interesting as they were derived from actual human photographs and thus followed Pollick's approach (Pollick, 2010) that humans can also fall in the UV when they sufficiently deviate from the typical human appearance. By contrast, android robots are often perceived to be robots because of their stiff posture and/or their visible mechanical parts. Therefore, we created artificial humans as a new category of stimuli that resemble android robots in their unnatural (synthetic) appearance but without visible mechanical parts or stiff posture and instead altered facial and head–body proportions and gray skin tone. Based on this design, artificial humans were thus conceptually closer to actual humans than android robots on a theoretically motivated humanlikeness continuum (Mori, 1970; Mori et al., 2012). We also found in separate pretests and in our main rating task that artificial humans were relatively less liked than android robots, likely because android robots offer relatively obvious cues regarding their status as machines, including stiff posture and visible mechanical parts, compared with the more subtle cues in artificial humans. We indicate in the Results that the main effects of defining the UV were robust when also including android robots in the analyses.

For analysis of choice data we used multiple logistic regression analysis. All regressions were performed at the random-effects level (i.e., regression coefficients were estimated separately for each subject and then entered into one-sample *t* tests at the group level to assess statistical significance). To assess the influence of the different rating differences we performed the following logistic regression:

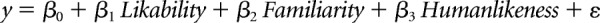
 where *y* is the observed choice on a single trial (0 for choice of first presented stimulus, 1 for choice of second presented stimulus), *likability* is the relative difference in rated likability between the first presented stimulus and second presented stimulus (calculated from the subject-specific and stimulus-specific likability rating given in the rating task), *familiarity* is the relative difference in rated familiarity between the first presented stimulus and second presented stimulus (calculated from the subject-specific and stimulus-specific familiarity rating given in the rating task), *humanlikeness* is the relative difference in rated humanlikeness between the first presented stimulus and second presented stimulus (calculated from the subject-specific and stimulus-specific humanlikeness rating given in the rating task), β_0_ is the constant term, β_1_ to β_3_ are the corresponding parameter estimates (regression coefficients), and ε is the residual. Accordingly, a subject's “decision variable” (cf. Fig. 5*D*) was modeled as follows:

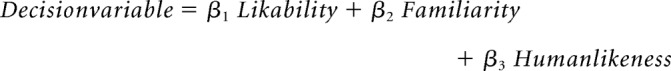
 Where *likability*, *familiarity*, *and humanlikeness* are the trial-by-trial relative rating differences as described above and β_1_ to β_3_ are the corresponding parameter estimates defined for each subject by the logistic regression described above. Thus, we modeled a subject's decision variable as a linear combination of subjectively weighted decision attributes.

For the analysis shown in Figure 5*D*, we binned the decision variable into equally populated bins (shown on the *x*-axis of Fig. 5*D*) and then determined the choice probability for each of these bins (as the number of observed choices for the first or second stimulus divided by the number of total choices in each bin); these data are represented by the circles in Figure 5*D*. The line in Figure 5*D* was obtained from fitting a logit function. For the analysis shown in Figure 5*E*, we focused on the five choice contrasts that compared humans without physical impairments to all other stimulus categories. For each choice contrast, we calculated the average absolute (unsigned) value of the decision variable, which we term “Δ *Decision variable*”. Thus, larger values for Δ *Decision variable* would indicate that on average, subjects evaluated the stimulus categories as very different in terms of their preference, and accordingly would have a strong preference for one category over the other.

##### fMRI data acquisition.

Functional MRI scanning was performed with a 7 T whole-body MRI system (Magnetom 7T, Siemens Healthcare) at the Erwin L. Hahn Institute for Magnetic Resonance Imaging, Essen, Germany. The system is equipped with the SC72 gradient system capable of 70 mT/m maximum amplitude and a slew rate of 200 mT/m/ms. For this experiment, the scanner was equipped with a 1 channel transmit/ 32-channel receive head coil (Nova Medical). For each participant, a T1-weighted high-resolution anatomical scan (same slice prescription as EPI) and magnetization-prepared rapid-acquisition gradient echo (MPRAGE) were acquired for registration purposes (repetition time (TR) = 2500 ms, echo time (TE) = 1.27 ms, inversion time (TI) = 1100 ms, flip angle = 7°, field of view (FOV) = 270 × 236 mm^2^, matrix = 394 × 345, sagittal plane, slice thickness = 0.7 mm, 256 slices with a noninterpolated voxel size of 0.7 × 0.7 × 0.7 mm^3^). For the acquisition of functional images, subjects were scanned in six subsequent sessions, each lasting about 12 min to acquire a total of 2022 volumes (331 – 343 volumes per session). In addition, subjects were scanned during two functional localizer tasks not used in the present paper, each lasting about 90 s. Whole-brain functional T2*-weighted EPI were acquired with a BOLD (blood oxygen level dependent) contrast-sensitive EPI sequence (Poser and Norris, 2009a,b) optimized for 7.0 T (slice thickness, 1.51 mm; 144 coronal slices; TR = 2000 ms; TE = 22 ms; flip angle, 14°; matrix, 170 × 170; FOV = 256 × 256 mm^2^, order of acquisition of slices: interleaved). As head coil array allows massive parallel imaging, the GRAPPA (generalized autocalibrating partially parallel acquisitions) algorithm was used with a reduction factor of *r* = 9 to reconstruct the undersampled *k*-space (Griswold et al., 2002). B0 field maps were acquired before the EPI sequence.

##### fMRI data analysis.

We performed the fMRI data analysis using statistical parametric mapping (SPM8; Wellcome Trust Centre for Neuroimaging, London). Preprocessing included realignment of functional data including motion correction, normalization to the Montreal Neurological Institute (MNI) coordinate system, and smoothing with a Gaussian kernel with full width at half maximum (FWHM) of 6 mm. A high-pass temporal filter with a cutoff period of 128 s was applied. General linear models (GLMs) assuming first-order autoregression were applied to the time course of activation in which event onsets were modeled as single impulse response functions convolved with the canonical hemodynamic response function. Time derivatives were included in the basis functions set. Linear contrasts of parameter estimates were defined to test specific effects in each individual dataset. Voxel values for each contrast resulted in a statistical parametric map of the corresponding *t* statistic. In the second (group random-effects) stage, subject-specific linear contrasts of these parameter estimates were entered into one-sample *t* tests, as described below, resulting in group-level statistical parametric maps. For parametric modulators, we used the standard SPM8 settings by which regressors are orthogonalized in the order they are entered into the design matrix. To test that our results are robust with respect to regressor orthogonalization, we performed ROI time course analyses (described below) in which regressors competed to explain variance in the absence of orthogonalization. Shared variances between our main variables of interest, computed within subjects and then averaged across subjects, were as follows: likability and familiarity, *R*^2^ = 0.39 (± 0.05); likability and humanlikeness, *R*^2^ = 0.26 (± 0.03); familiarity and humanlikeness, *R*^2^ = 0.36 (± 0.04); likability and human detection, *R*^2^ = 0.24 (± 0.03); familiarity and human detection, *R*^2^ = 0.24 (± 0.03); humanlikeness and human detection, *R*^2^ = 0.39 (± 0.03). Variance inflation factors (VIF) in our GLMs were within acceptable limits (mean VIF = 2.28 ± 0.19; Kutner et al., 2004). For the rating regressors in SPM analyses and ROI analyses we did not perform any weighting of the different rating variables but simply entered the mean-centered variables as regressors in the GLM. We estimated the following GLMs to test specific hypotheses as follows.

##### GLM 1.

**Figure 2.**
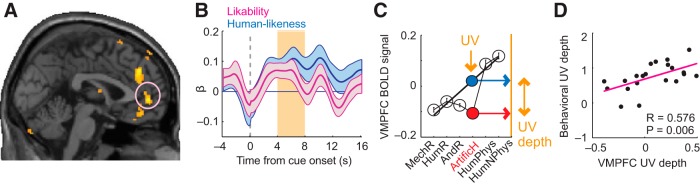
UV in VMPFC. ***A***, VMPFC signaled subjective likability. Activity during rating task reflected trial-by-trial likability ratings (*p* < 0.05, whole-brain corrected at cluster level; all statistical maps thresholded at *p* < 0.005, uncorrected for display purposes). ***B***, Integration of likability and humanlikeness. ROI regression of VMPFC activity on likability (*t*_(20)_ = 2.93, *p* = 0.008; one-sample *t* test in random-effects analysis) and humanlikeness (*t*_(20)_ = 3.45, *p* = 0.003). ***C***, VMPFC activity encodes UV reactions. As for likability ratings, VMPFC responses to artificial humans were lower than expected based on humanlikeness continuum. Black line: linear regression-fit of VMPFC activity to humanlikeness continuum from all stimuli except UV-relevant artificial humans (*r* = 0.268, *p* = 0.008, Pearson correlation). Blue point: predicted VMPFC response for artificial humans from linear fit; red point: measured response. UV depth defined as difference between predicted and measured response (deviation from linear fit: *t*_(20)_ = −2.97, *p* = 0.008, one-sample *t* test). ***D***, Neural UV depths matched behavioral UV depths. Linear regression of behavioral UV depths (Fig. 1*F*) on neural UV depths in VMPFC activity (*p* = 0.006; significant robust regression; mean-centered neural UV depths extracted from independently defined coordinates using leave-one-subject-out cross-validation).

**Figure 3.**
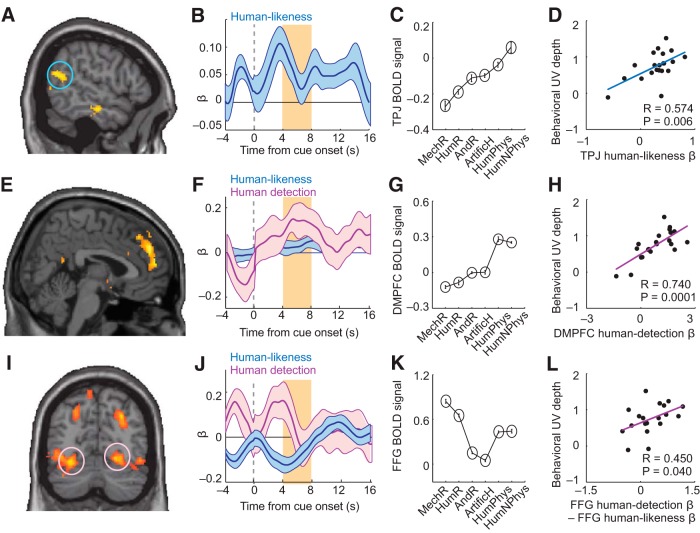
Linear and nonlinear humanlikeness coding. ***A***, Linear coding of subjective humanlikeness in TPJ. Activity reflected trial-by-trial humanlikeness ratings (*p* < 0.05, whole-brain corrected). ***B***, ROI regression of TPJ activity on humanlikeness (with likability and familiarity covariates; *t*_(20)_ = 3.47, *p* = 0.002, one-sample *t* test in random-effects analysis). ***C***, TPJ activity closely followed the humanlikeness continuum. ***D***, TPJ humanlikeness sensitivity reflected subjects' behavioral UV depths. Linear regression of behavioral UV-depth (cf. Fig. 1*F*) on TPJ humanlikeness βs (significant robust regression). ***E***, Stronger DMPFC activation for humans vs nonhumans shown by contrast analysis ([10 48 14], *z*-score = 4.78, *p* = 0.001, whole-brain corrected). ***F***, Regression of DMPFC activity on human detection (*t*_(20)_ = 2.26, *p* = 0.036) and humanlikeness (*p* = 0.603; nonsignificant likability and familiarity covariates). ***G***, Selective DMPFC response to humans. ***H***, Linear regression of behavioral UV depths on DMPFC human-detection βs (significant robust regression). ***I***, Negative humanlikeness coding in FFG (*p* < 0.05, whole-brain corrected). ***J***, Regression of FFG activity on human-detection (*t*_(20)_ = −2.26, *p* = 0.034) and humanlikeness (*t*_(20)_ = −3.56, *p* = 0.0019). Inset: significant β-difference for humanlikeness and human-detection. ***K***, FFG activity across stimulus categories. ***L***, FFG nonlinear humanlikeness sensitivity reflected behavioral UV depths (significant robust regression).

This GLM served three purposes: (1) to identify brain areas with rating-task activity related to rated likability, familiarity, and humanlikeness (see Figs. 2*A*, 3*A*,*I*); (2) to identify brain areas with choice-task activity related to the main decision variable and to rated decision confidence (see Figs. 6*A*, 7*A*); (3) to identify brain areas with higher activity in the choice task than in the rating task (see Table 2). For each subject, we estimated a GLM with the following regressors of interest: (R1) an indicator function for the stimulus onset during the rating task; (R2) R1 modulated by the trial-specific humanlikeness rating; (R3) R1 modulated by the trial-specific likability rating; (R4) R1 modulated by the trial-specific familiarity rating; (R5) an indicator function for the onset of the rating scales; (R6) an indicator function for the onset of the first stimulus during the choice task; (R7) an indicator function for the onset of the second stimulus during the choice task; (R8) R7 modulated by the trial-specific decision variable; (R9) R7 modulated by the trial-specific confidence rating; (R10) an indicator function for the onset of the choice phase; (R11) to (R16) the motion parameters resulting from the realignment preprocessing step as covariates of no interest; (R17) to (R22) six session constants. [Note that (R1) to (R16) were defined separately for each scanning session.]

##### GLM 2.

This GLM served to identify brain areas with choice-task activity related to the relative humanlikeness (see Fig. 6*H*). For each subject we estimated a GLM with the following regressors of interest: (R1) to (R6) as above; (R7) an indicator function for the onset of the second stimulus during the choice task; (R8) R7 modulated by the trial-specific confidence rating; (R9) R7 modulated by the trial-specific relative likability; (R10) R8 modulated by the trial-specific relative familiarity; (R11) R18 modulated by the trial-specific relative humanlikeness; (R12) an indicator function for the onset of the choice phase; (R13) an indicator function for the onset of the confidence rating scale; (R14) to (R20) the motion parameters resulting from the realignment preprocessing step as covariates of no interest; (R21) to (R26) six session constants. (Note that R1-R20 were defined separately for each scanning session.)

##### GLM 3.

This GLM served to identify brain areas with differential activity between specific stimulus categories (see Figs. 3*E*, 6*E*). For each subject, we estimated a GLM with the following regressors of interest: (R1) to (R6) indicator functions for the stimulus onsets during the rating task for each of the six stimulus categories defined above; (R7) an indicator function for the onset of the rating scales; (R8) to (R16) indicator functions for the onsets of the first stimulus during the choice task for each of the nine choice contrasts defined above; (R17) to (R25) indicator functions for the onsets of the second stimulus during the choice task for each of the nine choice contrasts defined above; (R26) an indicator function for the onset of the choice phase; (R27) to (R32) the motion parameters resulting from the realignment preprocessing step as covariates of no interest; (R33) to (R38) six session constants. [Note that (R1) to (R32) were defined separately for each scanning session.]

##### Functional connectivity analysis.

We assessed functional connectivity using the psychophysiological interaction (PPI) approach (Friston et al., 1997; Gitelman et al., 2003). For each subject, we first extracted eigenvariates for a 6 × 6 × 6 voxel cluster around a seed voxel based on the peak voxels identified by a contrast between the choice task and the rating task. The peak voxel used for each subject was determined using a leave-one-subject-out procedure by reestimating our second level analysis 21 times, each time leaving out one subject. Starting at the respective peak voxel for correlation with sequence length we selected the nearest peak in these cross-validation analyses. Time courses were deconvolved with the canonical hemodynamic response function (HRF) to construct a time series of neural activity in the ROI. The regressors were constructed using the standard deconvolution procedure as implemented in SPM8 (Gitelman et al., 2003). For each model, we calculated single-subject first-level contrasts for the PPI regressor (R1) that were then entered into a second level analysis by calculating a one-sample *t* test across the single subject coefficients. We estimated the following PPI GLMs.

##### PPI 1.

This GLM tested for differential coupling between brain areas as a function of stimulus category (humans vs nonhumans) in the rating task. A schematic summary of the results is shown in Figure 6*J*. The model contained the following regressors: (R1) a PPI regressor between the time series of activity in a seed brain area, extracted as just described, and a contrast between trials with human stimuli and nonhuman stimuli; (R2) the time series of activity in a seed brain area, extracted as just described; (R3) a contrast between human vs nonhuman stimuli; (R4–R9) the motion parameters resulting from the realignment preprocessing step as covariates of no interest; (R10-R15) six session constants. [Note that (R1) to (R9) were defined separately for each scanning session.] This model was estimated for the seed region DMPFC.

##### PPI 2.

This GLM tested for differential coupling between brain areas as a function of task (choice task vs rating phase). A schematic summary of the results is shown in Figure 6*J*. The model contained the following regressors: (R1) a psychophysiological interaction regressor between the time series of activity in a seed brain area, extracted as just described, and a contrast between choice task vs rating task; (R2) the time series of activity in a seed brain area, extracted as just described; (R3) a contrast between choice task vs rating task; (R4-R9) the motion parameters resulting from the realignment preprocessing step as covariates of no interest; (R10-R15) six session constants. [Note that (R1) to (R9) were defined separately for each scanning session.] This model was estimated for the seed regions TPJ, VMPFC, FFG, DMPFC, and amygdala.

##### Statistical significance *test*ing.

For all fMRI analyses, we report effects that survive correction for multiple comparisons across the whole brain using a significance level of *p* < 0.05 (family-wise error) at cluster level, imposed on maps that were displayed at cluster-defining threshold of *p* < 0.005 with minimum cluster size of *k* = 10 contiguous voxels. In addition, we used small volume correction (*p* < 0.05, cluster-level) in structures for which we had strong a priori hypotheses based on previous studies, including the VMPFC, ventral striatum, DMPFC, and amygdala. Small volume corrections were performed in spheres of 10 mm radius for cortical areas (VMPFC, DMPFC) and spheres of 6 mm radius for subcortical areas (ventral striatum, amygdala). The spheres were centered on specific coordinates reported in previous studies as follows. VMPFC [−2 40 −4] and ventral striatum [10 14 −4], taken from a meta-analysis of value-based decision making (Clithero and Rangel, 2014); amygdala: [18, −6, −22] taken from a previous study of value-based decisions using a similar experimental design to the present study but with different kinds of stimuli (Grabenhorst et al., 2013); DMPFC [2 44 36] taken from a previous study of social decision making (Wittmann et al., 2016). For DMPFC, we chose this particular study for our coordinate definition as it investigated DMPFC function in a task that required evaluating social others' performance to guide own decisions. We reasoned that similar processes might be engaged in our task, which required subjects to decide which agent would be capable of selecting a personal gift for them. Significant effects for humanlikeness and human–nonhuman contrast in DMPFC were also found when defining coordinates based on the Neurosynth meta-analysis database (Yarkoni et al., 2011), which localizes DMPFC at [0 56 22] using the search term “social.”

##### ROI analysis.

To ensure that statistical inference in our ROI analyses was not circular, we followed approaches used in previous studies (Behrens et al., 2008; Kriegeskorte et al., 2009; Esterman et al., 2010; Zangemeister et al., 2016). Specifically, we used a leave-one-subject-out method in which we reestimated a second-level analysis 21 times, each time leaving out one subject to define the ROI coordinates for the left-out subject. We then extracted the signal from the subject-specific coordinates defined in this way. Thus, the data which we used for the ROI analysis were independent from those used to define the coordinates for extracting the signal. Following data extraction we applied a high-pass filter with a cutoff period of 128 s. The data were then *z*-normalized, oversampled by a factor of 10 using sinc interpolation, and separated into trials to produce a matrix of trials against time. We generated separate matrices for each event of interest (rating trials, choice trials). We then fitted GLMs to each oversampled time point across trials separately in each subject.

For the rating task, our analysis strategy for ROI analyses was as follows: We first fitted a GLM containing as main regressors the three (parametrically varying) rating variables likability, humanlikeness and familiarity. We then tested whether the model fit was improved by inclusion of an additional binary human detection regressor that modeled the difference between human and nonhuman stimuli. We accepted this extended GLM if it yielded a better model fit as assessed with Akaike information criterion and significant human detection regressor. In the figures, we plot the standardized regression coefficients for significant regressors only; we report in the Results text which model provided the best fit and which regressors were significant. In addition to these regressors, the GLMs included motion parameters and session constants as covariates of no interest. This GLM analysis yielded one regression coefficient for each regressor for every oversampled time point in each subject. We entered individual-subject coefficients into one-sample *t* tests (random-effects analysis, *p* < 0.05) and calculated group averages and SEs for each time point across participants, yielding the across-subject effect size time courses shown in the figures. These mean effect size time courses are shown in Figures 2*B*; 3, *B*, *F*, and *J*; 6, *B*, *F*, and *H*; and 7, *B* and *C*.

To test for relationships between specific behavioral and neural effect sizes, we extracted neural effects sizes from individual subject's data using the leave-one-out procedure described above. We only tested for these relationships if a ROI showed a significant effect in the tested variable. We performed linear regression to produce the plots shown in the figures. We tested statistical significance using Pearson correlation. As the behavioral UV depths in the rating task and choice task entered these analyses multiple times, we performed a Bonferroni correction. Specifically, we obtained a *p*-value of *p* < 0.0125 for tests involving the behavioral UV depth in the rating task and a *p*-value of *p* < 0.0167 for tests involving behavioral UV depth in the choice task. The resulting effect size scatter plots are shown in Figures 2*D*; 3, *D*, *H*, and *L*; 6, *D* and *I*; and 7*D*.

## Results

### Behavioral UV reactions in the rating task

Subjects performed a psychophysical rating task in which they evaluated humans and different kinds of artificial agents on the key dimensions of the UV hypothesis, including humanlikeness, likability, and familiarity (Fig. 1*A*,*B*). Our approach to evoke UV reactions followed two concepts: First, we followed Mori's original hypothesis that likability increases with humanlikeness but sharply decreases for highly humanlike artificial agents (Mori, 1970; Mori et al., 2012). Second, we followed the argument that humans can also fall in the UV if they significantly deviate from typical human appearance or behavior (Pollick, 2010). Accordingly, we tested not only humanlike androids but, to elicit strong UV reactions, we designed “artificial humans” derived from photographs of humans with artificially altered facial features. These artificial or synthetic humans were more humanlike than typical androids but deviated from human appearance by having exaggerated smooth, flawless, unnatural faces and slightly unnatural proportions.

In total, we used four categories of artificial agents (Fig. 1*B*), based on independent pretests: (1) “artificial humans,” (2) “android robots,” (3) “humanoid robots,” and (4) “mechanoid robots.” We hypothesized that the humanlike artificial agents (in particular artificial humans, and androids) would fall in the UV, i.e., having lower likability ratings than predicted by a humanlikeness continuum (defined in the next paragraph), whereas less humanlike robots (humanoid and mechanoid robots) should lie outside the UV. As controls, we included photographs of humans with and without physical impairments, the former having presumed lower familiarity than the latter.

Stimulus categories differed significantly in rated likability (*F*_(1,20)_ = 46.19; *p* < 0.001; η^2^ = 0.698; repeated-measures ANOVA), familiarity (*F*_(1,20)_ = 54.82; *p* < 0.001; η^2^ = 0.733) and humanlikeness (*F*_(1,20)_ = 119.95; *p* < 0.001; η^2^ = 0.857). As predicted from the UV hypothesis, subjects evaluated the stimulus categories along a humanlikeness continuum (Fig. 1*C*, *r* = 0.980, *p* = 0.0006, linear regression): mechanoid and humanoid robots constituted the low end of this continuum, humans constituted the high end, and the UV-relevant artificial humans and android robots were rated intermediately humanlike. [Slightly lower ratings for humans with physical impairments, compared with those without physical impairments, were explained by lower familiarity: humanlikeness and likability ratings depended on familiarity ratings for humans with physical impairments (both R > 0.5, *p* < 0.05, Pearson correlation) but not for humans without physical impairments (*p* > 0.22)]. Within this observed humanlikeness continuum, we next measured UV reactions in likability.

Across all stimuli, likability tended to increase with humanlikeness (Fig. 1*D*). The key prediction of Mori's UV hypothesis is that, although likability generally increases with humanlikeness, highly humanlike artificial agents are less likable than predicted by a humanlikeness continuum, and thereby fall in the UV. Consistent with this prediction, likability ratings for artificial humans (and some specific androids) were lower than expected based on their rated humanlikeness (Fig. 1*D*). As proposed by the UV hypothesis (Mori, 1970) and found in previous work (depending on the appearance dimension that was varied, for instance, prototypicality (Burleigh et al., 2013)), likability data were well described by a cubic polynomial fit (*R*^2^ = 0.573, Fig. 1*D*), with consistent results in individual subjects' fits (mean *R*^2^ = 0.362 ± 0.03). A distinct UV effect was visible in the average likability of artificial humans, which was lower than expected based on the humanlikeness continuum (Fig. 1*E*). Thus, ratings across subjects and stimuli indicated the existence of a UV effect.

### Modeling the UV

To examine neural correlates of UV reactions, it was critical to first model and quantify the UV psychometrically within individual subjects. As we observed the most pronounced UV effect for artificial humans, we focused on this stimulus category (see Materials and Methods; including android stimuli yielded similar results).

We used a direct approach and quantified the UV effect as the extent to which likability ratings for artificial humans deviated from a linear fit of likability to humanlikeness, calculated across all other stimulus categories (“UV depth,” Fig. 1*F*). This approach captured Mori's original notion of the UV: an individual subject would have a stronger UV reaction (i.e., a deeper UV) if likability ratings for humanlike stimuli were lower than linearly predicted from that subject's humanlikeness ratings. Figure 1*F* illustrates the approach and resulting UV depth in one example subject, with further examples and across-subjects distribution shown in Figure 1*G*. The visible downward deflections of likability from linear humanlikeness fits (Fig. 1*F*,*G*, compare red and blue data points, indicating observed likability and likability predicted from humanlikeness, respectively) implied that artificial humans were less liked than expected from a linear humanlikeness function.

We found robust behavioral UV reactions in the rating data. Despite considerable interindividual variation, UV depths for artificial humans were significantly larger than predicted from humanlikeness (indicating lower likability than predicted; *p* = 3.47 × 10^−8^, one-sample *t* test). UV depth was significant in 18 of 21 individual subjects (*p* < 0.05, one-sample *t* tests). (Weaker but significant UV reactions were found for familiarity (*p* = 1.9 × 10^−4^) but, as expected, not for humanlikeness (*p* = 0.117)). A significant UV effect was also found when including both artificial humans and androids (*p* = 6.1 × 10^−6^). As controls, UV depths were nonsignificant for stimuli not hypothesized to fall in the UV, (e.g., humans with physical impairments or humanoid robots; both *p* > 0.15, one-sample *t* tests), for which likability was well predicted by a linear humanlikeness fit.

We confirmed the robustness of these results by an alternative approach to model the UV based on regression residuals. Within subjects, we fitted six linear regressions of likability ratings on humanlikeness ratings (cf. Fig. 1*D*), each time leaving out the data from one of the six stimulus categories. We then applied the estimated regression coefficients on the data of the left-out category to predict likability from humanlikeness and noted the regression residuals (i.e., the variance in likability not explained by humanlikeness given the specific regression coefficients). According to the UV hypothesis, these residuals should be significantly more negative for the UV-relevant artificial humans (and possibly android robots) compared with other stimulus categories. Indeed, residuals for artificial humans were significantly smaller (more negative) than those for all other agents (all comparisons *p* < 0.005, *t* test, corrected for multiple comparisons), while the residuals for androids were significantly smaller than those for humanoids and humans without physical disabilities (both *p* < 0.005). The UV effect quantified within each subject based on these residuals was highly correlated with our main measure of the UV effect (*r* = 0.7486, *p* = 9.4 × 10^−5^, Pearson correlation). Thus, this procedure provided additional validation for the existence of a UV effect in the behavioral data.

Thus, behavioral data confirmed the existence of a UV reaction in our stimulus set and validated the rating task to search for neural correlates of subjective UV reactions. We next investigated how neural systems might transform a linear humanlikeness function into a nonlinear UV function.

### A neural UV in VMPFC

To locate neural correlates of UV reactions, we regressed stimulus-evoked activity in the rating task on trial-by-trial rated likability, which was the key rating to reflect the UV (shown above). Likability evaluations were encoded in typical reward areas, including VMPFC (Fig. 2*A*,*B*). Additionally, VMPFC activity reflected humanlikeness ratings (Fig. 2*B*). Because likability and humanlikeness were covariates, these variables accounted for different activity-components. Familiarity did not explain VMPFC activity. (All ROI analyses used leave-one-subject-out cross-validation to identify subject-specific, independent ROI coordinates, ensuring unbiased analysis; Kriegeskorte et al., 2009; Esterman et al., 2010). ROI regressions were performed without orthogonalization. All three ratings were included as regressors; in addition, we tested in a second GLM whether a binary human detection contrast would improve model fit (see Materials and Methods).] We tested whether the GLM in the ROI analysis was improved by inclusion of a binary “human detection” regressor that contrasted human and nonhuman stimuli. (For this contrast, nonhuman agents involved mechanoid, humanoid, android and artificial-human stimuli.) Inclusion of this human detection regressor did not change the significant effects for likability and humanlikeness shown in Figure 2*B*; although the human detection regressor itself showed a significant effect on VMPFC activity, this occurred quite late outside our primary analysis window (*p* = 0.033, one-sample *t* test at 9 s poststimulus onset). Thus, VMPFC integrated the two key UV-dimensions likability and humanlikeness, suggesting a role in UV reactions.

If a brain area signaled the key UV dimension likability, it might also explicitly represent the UV reaction. (In an “explicit” UV representation, activity patterns across stimuli should match the prototypical UV shape.) Consistent with this notion, VMPFC activity across stimuli closely resembled the behavioral UV reaction (Fig. 2*C*): activity increased approximately linearly according to humanlikeness for most stimuli, but responses to artificial humans were significantly lower than expected from a linear humanlikeness fit (Fig. 2*C*; *p* = 0.008, one-sample *t* test). VMPFC activity thus followed the typical UV shape with selectively lower activity for highly humanlike artificial agents. Across subjects, neural UV depths (derived from individual subjects' VMPFC activities) matched subjects' behavioral UV reactions (Fig. 2*D*; *r* = 0.576, *p* = 0.006, Pearson correlation, Bonferroni corrected). When calculating this across-subjects effect with the alternative UV-quantification based on regression residuals (described above), the relationship with neural UV depths in VMPFC was weaker (*r* = 0.41, *p* = 0.062). Accordingly, the relationship to individual differences should be considered exploratory.

Thus, VMPFC activity integrated the key UV dimensions likability and humanlikeness to an explicit representation of the UV reaction. The strength of this representation partly explained individual differences in behavioral reactions toward artificial agents.

### Linear and nonlinear humanlikeness signals as neural basis for the UV

We next searched for activities related to the UV dimension humanlikeness. We reasoned that different types of humanlikeness signals might constitute neural inputs required for transforming the psychophysical humanlikeness continuum (Fig. 1*C*) into the nonlinear UV representation observed in VMPFC (Fig. 2*C*).

Neural responses to human and artificial stimuli in TPJ, DMPFC, and part of fusiform gyrus (FFG) were related to humanlikeness ratings (Fig. 3, Table 1). TPJ activity showed a positive linear relationship with humanlikeness (Fig. 3*A–C*): it gradually increased across stimuli and faithfully reflected the humanlikeness continuum. Likability and familiarity did not show significant effects on TPJ activity; a human detection regressor was not significant and its inclusion did not affect the significance of the humanlikeness regressor. TPJ thus provided a parametric, linear humanlikeness signal—the most basic element of the UV hypothesis.

**Table 1.** Rating task analyses

Effect	Area	*x*	*y*	*z*	*z*-score	*p*
Human-likeness PM, positive	TPJ	56	−60	20	3.53	0.029
	DMPFC	4	40	42	3.14	0.018 sv
Human-likeness PM, negative	FFG	26	−66	−12	4.22	0.001
	Occipital gyri	−20	−102	2	5.54	0.001
	Middle frontal gyrus	−48	36	24	3.90	0.001
Likability PM, positive	VMPFC	12	48	8	4.00	0.001
	Striate area	30	−98	18	3.87	0.002
	Ventral striatum	12	12	−10	3.10	0.043 sv
Likability PM, negative	—					
Familiarity PM, positive	Ventral striatum	10	14	−4	3.40	0.025 sv
Familiarity PM, negative	—					

Results are whole-brain corrected, *p* < 0.05, cluster level; maps thresholded at *p* < 0.005, extent threshold 10 voxels.

PM, Parametric modulator; C, contrast; sv, small-volume correction, based on predefined coordinates (see Materials and Methods). *x*, *y*, and *z* are the coordinates in MNI space.

By contrast, humanlikeness coding in DMPFC was more complex. Although we found a significant relationship with parametric humanlikeness (Table 1, GLM1), a binary contrast showed significantly stronger activation by human agents compared with nonhuman agents (Fig. 3*E*; GLM3, contrasting mechanoid, humanoid, android robots and artificial humans with both human stimulus categories). Detailed ROI analysis indicated that neural activity in this DMPFC area was best explained by a human detection contrast as follows. Across stimuli, DMPFC activity followed the humanlikeness continuum for nonhuman agents but then sharply increased for human agents (Fig. 3*E–G*). We modeled this activity with a “human detection” regressor (Fig. 3*F*, *a* dummy variable distinguishing human from nonhuman stimuli) in addition to linear humanlikeness (improvement in GLM fit was assessed by Akaike Information Criterion). (Although Figs. 2*C* and 3*G* may look similar, it is important to note that these data are averaged across trials and subjects; our ROI analysis within each subject indicated that, whereas VMPFC activity was best explained by joint likability and human likeness coding, DMPFC activity was best explained by a human detection regressor.) Thus, DMPFC activity emphasized differences between human and nonhuman stimuli, suggesting a role in distinguishing human from artificial agents.

FFG exhibited a third type of humanlikeness signal. It showed a negative parametric relationship with humanlikeness selectively for nonhuman stimuli and an average response to human stimuli (Fig. 3*I–K*, modeled in the same way as DMPFC activity). Both humanlikeness and human detection explained significant variance in FFG activity (Fig. 3*J*, inset). Thus, FFG combined a human detection response with negative humanlikeness coding selectively for artificial agents.

If TPJ, DMPFC and FFG contributed to UV reactions, their sensitivity to humanlikeness should reflect individuals' UV depths. Indeed, all three areas encoded humanlikeness more strongly for subjects with stronger UV reactions (Fig. 3*D*,*H*,*L*). [These relationships were significant for humanlikeness coefficients (*p* < 0.05, Pearson correlation); in DMPFC, the effect was strongest for human-detection and in FFG for differential humanlikeness.] Similar effects were not found in control analyses with non-UV stimulus categories (*p* > 0.6). We note that the effect in Figure 3*L* did not survive Bonferroni correction for multiple comparisons; accordingly, we treat this result as an exploratory finding.

Thus, TPJ, DMPFC, and FFG encoded distinct linear and nonlinear humanlikeness signals that were related to individuals' UV reactions.

### Modeling the neural UV from humanlikeness signals

These data suggest a possible information-processing sequence, whereby a linear humanlikeness code in TPJ is transformed to nonlinear humanlikeness codes in DMPFC and FFG and eventually to an explicit UV representation (i.e., nonlinear likability) in VMPFC. Specifically, the observed FFG signal seemed to track the proximity to a human–nonhuman boundary (decreasing activity selectively for non-humans), which seems suited for determining the UV-typical likability-drop for the most humanlike artificial agents. The DMPFC human-detection signal might be relevant for setting up such selective humanlikeness signaling.

**Figure 4.**
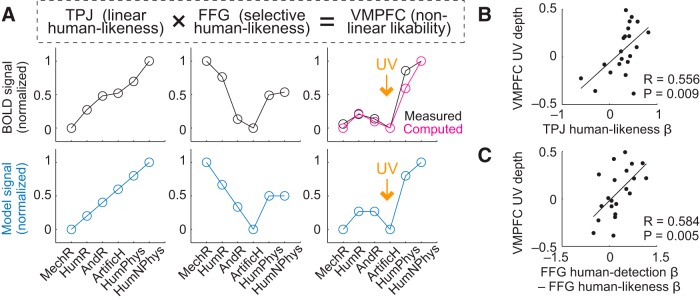
Constructing the UV from linear and nonlinear humanlikeness signals. ***A***, Combining humanlikeness signals in TPJ and FFG to construct nonlinear likability in VMPFC. Top, Measured activity patterns (across subjects) in TPJ, FFG, and VMPFC. Multiplicative combination of measured TPJ and FFG signals (“computed”) approximated the observed neural UV in VMPFC (“measured”). Bottom, Modeled signals. TPJ activity modeled by linear humanlikeness function; FFG activity modeled as inverse, linear humanlikeness function (negative linear relation across hypothesized humanlikeness continuum), selectively for nonhumans, with undifferentiated (average) response to human stimuli; VMPFC activity modeled as multiplicative combination of these signals. ***B***, ***C***, Relation between neural humanlikeness sensitivities and neural UV depth in VMPFC across subjects. ***B***, Linear regression of TPJ humanlikeness on VMPFC UV depth (significant robust regression). ***C***, Linear regression of FFG humanlikeness on VMPFC UV depth (significant robust regression).

Consistent with these notions, a simple multiplicative integration of linear and nonlinear humanlikeness signals in TPJ and FFG approximated the UV-related activity pattern in VMPFC (Fig. 4*A*). Such multiplicative signal integration is biologically plausible and routinely used in models of neural multisensory integration (Peña and Konishi, 2001; de Araujo et al., 2003; Small et al., 2004; Stein and Stanford, 2008). Across subjects, the strengths of the humanlikeness components in TPJ and FFG were related to the neural UV-effect in VMPFC (Fig. 4*B*,*C*), suggesting these combined signals were relevant for the observed UV effect in VMPFC.

Thus, the neural UV code in VMPFC could be approximated by multiplicative integration of humanlikeness signals from TPJ and FFG. We next tested in a second experiment how these activities were related to behavioral choices toward artificial agents.

### UV reactions guiding decision making

**Figure 5.**
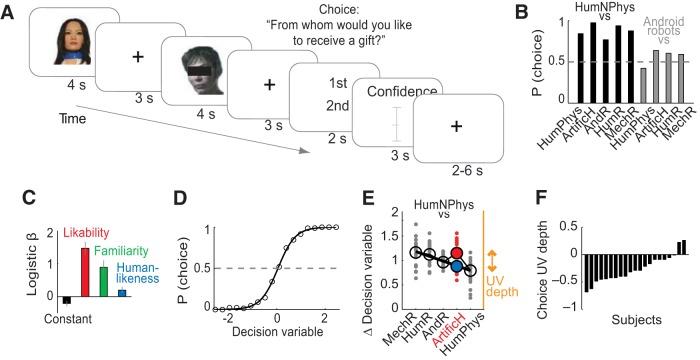
Decision making to accept or reject artificial agents. ***A***, Choice task. Subjects viewed sequential stimuli and decided from whom they would like to receive a gift (example from categories android robots (first screen) and artificial humans (second screen); shown without black bar during the experiment. ***B***, Choice behavior. Probability for gift-acceptance from humans (blacks) and androids (gray) over other stimulus categories. ***C***, Modeling choices with logistic regression. β weights of relative likability, familiarity and humanlikeness (all *p* < 0.002, ones-sample *t* tests). ***D***, Psychometric function: Relation between decision variable and choice probability. Relative differences in likability, familiarity and humanlikeness were weighted for individual subjects, based on subject-specific logistic regression, and summed to form a decision variable. Choice probabilities calculated for equally populated decision-variable bins and fitted with logit function. ***E***, Defining UV depth for the choice task, based on decision variable. Unsigned (absolute) differences in decision variable between stimulus categories (Δ Decision Variable, derived from stimulus-specific ratings during rating task; data for all subjects). Black line: linear regression-fit to humanlikeness continuum for all conditions (gray data) except choices involving artificial humans (red data). Blue point: predicted decision variable for artificial humans from linear fit; red point: observed decision variable. Choice UV depth defined as difference between predicted and observed decision variable. ***F***, Choice UV depth across subjects. The procedure in ***F*** defined UV depths for all subjects (*p* = 1.1 × 10^−4^, one-sample *t* test).

In a second experimental task (Fig. 5*A*), subjects viewed sequential human and artificial agents and chose from whom they would rather receive a personal gift. We instructed subjects that each human and artificial agent had selected a gift from an option set, and that they would receive one of these gifts based on their choices for the different agents during the experiment. Actual gift choices were unknown to the subjects; they had to decide whom they would trust to select an attractive gift. We reasoned that this situation likely encouraged subjects to compare humans and artificial agents on UV-relevant dimensions.

When choosing whether to prefer a gift from a human or artificial agent, subjects typically (but not always) preferred humans (Fig. 5*B*, black bars). Choice probabilities were more variable when choosing between artificial agents (gray bars). We next sought to explain subjects' choices in terms of the separately made ratings of key UV dimensions.

In value-based choice, a decision-maker integrates relevant information to a “decision variable” (a weighted composite of decision factors) to compare and decide between different options. Accordingly, we reasoned that weighted, comparative valuations of likability, humanlikeness and familiarity guided subjects' choices. We defined trial-specific relative differences in likability, humanlikeness and familiarity based on individual subjects' stimulus ratings (from the rating task). Logistic regression showed that relative likability, familiarity and humanlikeness were significant predictors for subjects' choices: subjects were more likely to accept a gift from an individual they considered relatively more likable, familiar, and humanlike (Fig. 5*C*, all regression coefficients *p* < 0.0002, one-sample *t* tests). The same result was found when considering only choices involving UV-relevant artificial humans and androids (all coefficients *p* < 0.0009, one-sample *t* tests). On average, logistic regression based on relative ratings provided correct choice classification for 85.7% of trials (mean across subjects, ± 0.89, SEM) and resulted in a pseudo-*R*^2^ of 0.53 (mean across subjects, ± 0.02, SEM). Choice probabilities were thus well described by a subject-specific decision variable, defined as a weighted sum of relative likability, familiarity, and humanlikeness (Fig. 5*D*).

To quantify the UV in the choice task, we examined the decision variable for specific stimulus categories. For this analysis and subsequent neural analyses, we focused on the unsigned decision variable i.e., the absolute value difference. In value-based decisions, neural decision signals often reflect this absolute value difference between choice options; (Heekeren et al., 2004; Rolls et al., 2010a,b; Hunt et al., 2012; Grabenhorst et al., 2013), which is usually interpreted as the signature of a competitive decision mechanism.

Similar to likability ratings (Fig. 1*F*), the decision variable followed a humanlikeness continuum: the more two stimulus categories differed in humanlikeness, the more they also differed in the decision variable (Fig. 5*E*; *r* = −0.533, *p* = 1.8 × 10^−7^, Pearson correlation), with highest differences for humans versus mechanoid robots and smallest differences for humans with and without physical impairments. (A high difference in the decision variable between stimuli indicated clear choice preference between these stimuli.) However, consistent with the UV hypothesis, the difference in the decision variable between humans and artificial humans was significantly larger than expected from a humanlikeness continuum (Fig. 5*F*; *t*_(20)_ = 4.66, *p* = 1.52 × 10^−4^, one-sample *t* test), which matched the UV reaction observed in likability ratings (Fig. 1*D*). This upward deflection from the linear humanlikeness fit (Fig. 5*E*, difference between red and blue data points, indicating actual difference in decision variable and difference in decision variable estimated from linear humanlikeness fit, respectively) implied a greater difference than expected if the decision variable followed a humanlikeness continuum. This result quantified the UV during choices in terms of individual subjects' decision variable. Choice UV effects were not found for control stimuli (humanoid robots; *p* = 0.505, one-sample *t* test).

Thus, choices between humans and artificial agents were based on a decision variable that reflected subjects' individual UV reactions. These data validated the choice task for examining neural correlates of decision making in the context of the UV hypothesis.

### Neural UV components during decisions

A contrast showed that areas involved in the rating task were even more strongly activated in the choice task, including VMPFC, DMPFC, and TPJ (Table 2). To examine neural UV components during decisions, we regressed choice-task activity (responses to humans and artificial agents at time of the second stimulus on each trial) on individual subjects' decisions variables; that is, the weighted sums of relative likability, familiarity and humanlikeness.

**Table 2.** Choice task analyses

Effect	Area	*x*	*y*	*z*	*z*-score	*p*
Decision variable PM, positive	VMPFC	0	34	6	3.60	0.019 sv
	TPJ	68	−34	44	3.30	0.030
	Caudate nucleus	−12	24	12	4.53	0.001
	Occipital gyri	20	−90	30	4.50	0.001
	Occipital gyri	18	−54	12	4.40	0.010
	Cingulate gyrus	6	−42	46	3.77	0.003
	Occipital gyri	−22	−60	24	3.72	0.042
Decision variable PM, negative	Amygdala	16	−8	−20	3.49	0.022 sv
	Intraparietal sulcus	−32	−56	46	4.48	0.001
	Superior frontal gyrus	−10	−4	72	4.43	0.001
	Middle frontal gyrus	−42	8	36	4.09	0.001
	Middle frontal gyrus	34	4	32	3.86	0.015
Relative human-likeness PM, positive	TPJ	54	−64	8	4.50	0.001
Relative human-likeness PM, negative	Occipital gyri	24	−98	8	5.99	0.001
	Occipital gyri	−24	−94	−2	4.74	0.001
Choice > rating, C	VMPFC	4	44	2	4.00	0.001
	TPJ	54	−64	26	5.51	0.001
	TPJ	−52	−58	28	5.71	0.001
	Amygdala	22	6	−26	4.32	0.001
	Ventral striatum	12	10	−6	3.43	0.025 sv
	Precuneus	2	−68	24	6.11	0.001
	Superior frontal gyrus	10	22	66	5.22	0.001
	Planum polare	34	14	−22	4.93	0.001
	Middle temporal gyrus	68	−34	−6	4.53	0.001
	Cingulate gyrus	0	−14	34	4.11	0.004
Rating > choice, C	Occipital gyri	34	−88	12	6.46	0.001
	FFG	−32	−80	−18	5.68	0.001
	Precentral gyrus	−26	−16	76	4.81	0.001
	Middle frontal gyrus	−42	22	26	4.66	0.002

Results are whole-brain corrected, *p* < 0.05, cluster level; maps thresholded at *p* < 0.005, extent threshold 10 voxels.

PM, Parametric modulator; C, contrast; sv, small-volume correction, based on predefined coordinates (see Materials and Methods). *x*, *y*, and *z* are the coordinates in MNI space.

**Figure 6.**
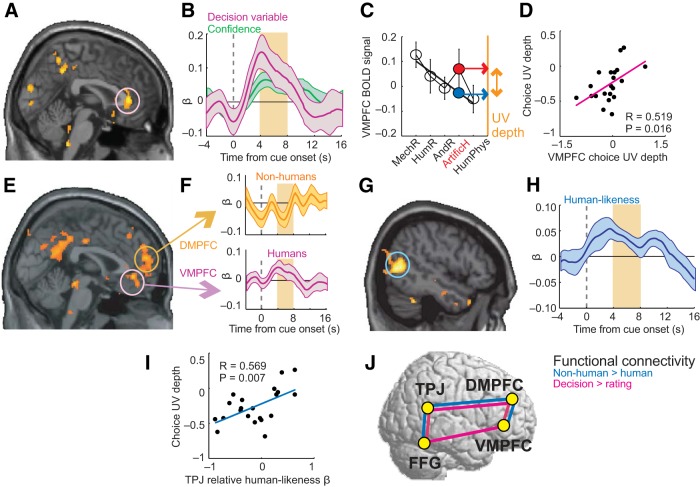
Neural UV components during decision making. ***A***, VMPFC activity during gift-choices between humans and artificial agents coded subjects' decision variable (*p* < 0.05; small-volume correction). Decision variable used as parametric modulator was the sum of subjectively weighted relative likability, familiarity and humanlikeness, which explained choices (cf. Fig. 5*E*, *F*). ***B***, ROI regression of VMPFC activity on decision variable (*t*_(20)_ = 2.25, *p* = 0.0356; one-sample *t* test, random-effects analysis) and rated confidence (*t*_(20)_ = 3.48, *p* = 0.002). ***C***, VMPFC activity during choices reflects UV. VMPFC responses during choices involving artificial humans deviated from expected humanlikeness continuum. Black line: linear regression-fit of activity on relative humanlikeness for all stimuli except artificial humans (*r* = −0.248, *p* = 0.023, Pearson correlation). Blue point: predicted response for artificial humans from linear fit; red point: measured response. ***D***, Linear regression of behavioral choice UV depth (defined in Fig. 5*E*) on neural UV depth derived from VMPFC activity (*p* = 0.016; significant robust regression). ***E***, Stronger activation in VMPFC ([-6 40 −6], *z* = 4.21) and DMPFC ([0 56 18], *z* = 3.81) during choices involving humans contrasted with choices without humans (both *p* < 0.001, whole-brain corrected). ***F***, DMPFC coded decision variable specifically for choices involving nonhumans (*t*_(20)_ = −2.45, *p* = 0.0294, one-sample *t* test); VMPFC coded decision variable specifically for choices involving humans (*t*_(20)_ = 2.139, *p* = 0.044, one-sample *t* test). ***G***, TPJ activity reflected the humanlikeness component of the decision variable (*p* < 0.05, whole-brain corrected). ***H***, ROI regression of TPJ activity on relative humanlikeness (*t*_(20)_ = 3.678, *p* = 0.0015, one-sample *t* test). ***I***, Behavioral UV depths matched TPJ humanlikeness βs (robust regression). ***J***, Functional connectivity. Psychophysiological interactions identified pairs of brain regions with stronger activity-correlations during choices than ratings (magenta connections) or stronger activity-correlations for nonhumans compared with humans (blue connections, rating task). Summary figure based on whole-brain-corrected statistical maps.

Activity in VMPFC tracked subjects' decision variable, with stronger activity for larger unsigned value differences (Fig. 6*A*,*B*). Similar effects occurred in other areas implicated in decision-related valuations (Bartra et al., 2013), including striatum and posterior cingulate cortex (Table 2). VMPFC encoding of the decision variable was not accounted for by subjective decision difficulty: confidence ratings (a common measure of subjective difficulty) explained separate VMPFC activity components (Fig. 6*B*). Across decision categories, VMPFC activity followed a humanlikeness continuum but deviated significantly for choices involving UV-relevant artificial humans, thereby matching the psychophysical UV (Fig. 6*C*). (Note that Figure 6*C* shows VMPFC activity when subjects chose whether to receive a gift from humans compared with different agents, labeled on *x*-axis.) The significant deviation from linear fit for choices between humans and artificial humans (Fig. 6*C*, compare red and blue points) suggested stronger VMPFC activation (indicating more disparate value difference) than expected from a humanlikeness continuum, specifically for artificial agents that elicited UV reactions. Across subjects, this neural UV correlated with subjects' behavioral UV depths (Fig. 6*D*). Thus, VMPFC encoded the behaviorally important decision variable in close relation to individual differences and reflected UV reactions during decision making.

Previous studies showed separate functions of VMPFC and DMPFC during choices for self and others (Nicolle et al., 2012; Wittmann et al., 2016) and during evaluations of similar and dissimilar others (Mitchell et al., 2006). We therefore examined decision activity of these areas for choices involving humans (vs other stimulus categories) and choices not involving humans (choices among artificial agents). Contrasting decision-trials involving humans and nonhumans showed stronger activation in both DMPFC and VMPFC (Fig. 6*E*). Given this differential activation, we performed a ROI regression on DMPFC and VMPFC activity in which the decision variable was modeled separately for choices involving humans and choices involving nonhumans. This analysis revealed selective coding of the decision variable in DMPFC for choices involving nonhumans (Fig. 6*F*, top, negative coding scheme). By contrast, VMPFC coded the decision variable specifically for choices involving humans (Fig. 6*F*, bottom, positive coding scheme). Thus, VMPFC and DMPFC coded subjects' decision variable selectively, and complementarily, in a human–nonhuman frame of reference.

Regressing decision activity specifically on relative humanlikeness, a subcomponent of the decision variable important in UV reactions, showed a significant effect in the TPJ region that also encoded humanlikeness during ratings (Fig. 6*G*,*H*). Across subjects, TPJ's sensitivity to humanlikeness was related to behavioral UV reactions (Fig. 6*I*), suggesting behavioral relevance for subjects' choices. Additionally, similar to the rating task, ROI regression on FFG activity showed a significant human-detection contrast (*t*_(20)_ = 2.27, *p* = 0.033, one-sample *t* test) and negative humanlikeness coding (*t*_(20)_ = 4.39, *p* = 0.0003, one-sample *t* test).

We examined functional connectivity with PPI analyses. We found that TPJ was more strongly connected with both DMPFC and FFG during choices compared with ratings (Fig. 6*J*, magenta, Table 3), reflecting these areas' observed common humanlikeness encoding. Functional connections also existed between VMPFC and both DMPFC and FFG (Fig. 6*J*, magenta), but we found no direct coupling between VMPFC and TPJ. Thus, areas implicated in valuations of humanlikeness, likability, and subjects' decision variable interacted functionally during decision making.

**Table 3.** PPI analyses

Effect	Area	*x*	*y*	*z*	*z*-score	*p*
PPI nonhuman > human, DMPFC seed	VMPFC	6	34	10	3.82	0.006
	DMPFC	4	46	32	4.18	0.006
	TPJ	46	−70	34	3.97	0.019
	Posterior cingulate cortex	−8	−48	36	4.19	0.001
PPI nonhuman > human, FFG seed	TPJ	50	−36	26	3.64	0.020
PPI Choice > rating, VMPFC seed	FFG	14	−70	−10	5.02	0.001
	Cingulate gyrus	−4	18	40	4.22	0.001
	Caudate nucleus	24	−19	26	4.15	0.001
PPI Choice > rating, FFG seed	TPJ	50	−38	28	4.32	0.001
	FFG	−18	−64	−6	4.22	0.028
	Precuneus	0	−78	28	3.98	0.012
PPI Choice > rating, TPJ seed	DMPFC	0	46	38	3.52	0.001
	Occipital gyri	10	−102	18	4.95	0.021
	Superior frontal gyrus	−8	20	66	4.82	0.001
PPI Choice > rating, DMPFC seed	VMPFC	−2	44	10	4.11	0.001
	Cingulate gyrus	−14	54	30	4.39	0.005
	Occipital gyri	14	−100	6	4.12	0.002

Results are whole-brain corrected, *p* < 0.05, cluster level; maps thresholded at *p* < 0.005, extent threshold 10 voxels.

PM, Parametric modulator; C, contrast; sv, small-volume correction, based on predefined coordinates (see Materials and Methods). *x*, *y*, and *z* are the coordinates in MNI space.

### Amygdala signals for rejecting human and nonhuman agents

The amygdala is a subcortical structure involved in emotion and social information processing (Phelps and LeDoux, 2005; Adolphs, 2010) that contributes to decision making (Seymour and Dolan, 2008; Grabenhorst et al., 2012, 2013, 2016; Zangemeister et al., 2016). Contrast analysis suggested amygdala engagement in the choice task (Table 2). We therefore examined its role in UV-related decisions.

**Figure 7.**
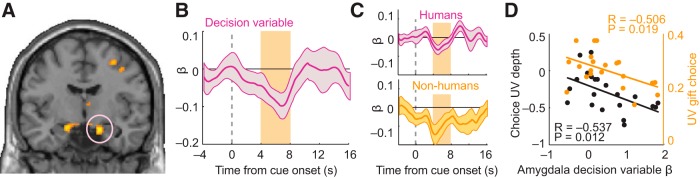
Amygdala rejection-signals for humans and artificial agents. ***A***, Amygdala choice activity coded subjects' decision variable (*p* < 0.05; small-volume correction on predefined amygdala coordinates). ***B***, ROI regression showed negative amygdala-coding of decision variable (*t*_(20)_ = −3.05, *p* = 0.006, one-sample *t* test). ***C***, Significant coding of decision variable for choices involving humans (*p* = 0.0173, one-sample *t* test) and choices without humans (*p* = 0.005). ***D***, Amygdala sensitivity to decision variable predicted behavioral UV depths (black data) and gift rejections from artificial agents (orange data). Linear regressions of choice UV depths (*p* = 0.012, robust regression) and probability of gift acceptance from artificial humans and androids (*p* = 0.019, robust regression) on amygdala βs.

Similar to VMPFC, the amygdala encoded subjects' decision variable (Fig. 7*A*), but with a negative coding scheme whereby higher activity indicated lower decision values (Fig. 7*B*) and without additional confidence-coding (*p* = 0.741, one-sample *t* test). Unlike VMPFC and DMPFC, the amygdala encoded subjects' decision variable regardless of whether decisions involved humans or nonhuman agents (Fig. 7*C*,*D*), suggesting a context-invariant decision signal. As for VMPFC, amygdala's encoding of the decision variable was related to individual differences in UV reactions (Fig. 7*E*, black data). The amygdala was distinct as its coding was directly related to subjects' choice probabilities: subjects with higher amygdala decision-sensitivity were less likely to accept gifts from artificial agents (Fig. 7*E*, orange data). Similar relationships were not found for VMPFC (*p* = 0.777, Pearson correlation) or human gift acceptance (*p* = 0.317). The amygdala was not functionally coupled to other areas, except for a nonsignificant effect in DMPFC. Thus, the amygdala encoded subjects' decision variable, and this coding partly explained gift refusal from UV-relevant artificial agents.

## Discussion

We investigated neural activity when subjects evaluated different attributes of human and artificial agents and made personal decisions about these agents. We found that stimulus-evoked activity in VMPFC, a key valuation structure (Chib et al., 2009; Hare et al., 2010; Wunderlich et al., 2010; Bartra et al., 2013), matched the characteristic nonlinear shape of the UV reaction (Mori, 1970; Mori et al., 2012): VMPFC responses to human and artificial agents increased according to the psychophysically measured humanlikeness continuum but were markedly decreased for the most humanlike artificial agents (“artificial humans”), which fell in the psychophysically measured UV. Across subjects, the depth of this neural UV—formalized as a deviation from a linear humanlikeness fit—explained individual differences in behavioral UV reactions. VMPFC was a candidate site for computing the UV response, as it jointly encoded humanlikeness and likability signals. Consistently, during decision making, VMPFC activity reflected the UV in terms of a decision variable (derived from subjectively weighted decision attributes) that guided subjects' choices between humans and artificial agents. These data demonstrate a surprisingly direct neural representation of the UV as a nonlinear value function and provide neurobiological evidence for the key prediction of the UV hypothesis that the UV derives from integrated humanlikeness and likability evaluations.

A distinct set of brain areas implicated in social information processing encoded the humanlikeness dimension underlying the UV hypothesis. A humanlikeness continuum was faithfully and linearly encoded by neural responses to artificial agents in the TPJ, consistent with this area's roles in agency-detection (Mar et al., 2007), belief-attribution (Saxe and Wexler, 2005; Carter and Huettel, 2013; Vogeley, 2017) and learning about others (Behrens et al., 2008; Hampton et al., 2008; Boorman et al., 2013). TPJ thus represented the most basic element of the UV hypothesis, humanlikeness, on a linear scale of neural activity. Such a linear neural representation is computationally useful (Rolls and Treves, 1998), as it provides a versatile basis for deriving other, nonlinear neural representations of related variables, as observed in DMPFC, FFG, and VMPFC. Thus, TPJ likely provided important inputs for generating neural UV representations, as also suggested by the observed relationship to individual differences.

By contrast, the DMPFC, a region implicated in mentalizing (Saxe and Wexler, 2005; Amodio and Frith, 2006), responded particularly strongly to human agents and coded humanlikeness by a nonlinear, step-like function that emphasized differences between humans and nonhumans. Previous studies observed differential DMPFC activity when making choices for self or others (Nicolle et al., 2012), when tracking performance for self or other (Wittmann et al., 2016), and when attributing mental states to others (Mitchell et al., 2006). The present data extend these observations toward a distinction between human and nonhuman social others. Such human-detection activity might be critical in setting up a UV effect as it could set a threshold of “humanlike but not genuinely human” that would trigger the characteristic drop in likability.

The FFG encoded an additional candidate input for computing UV reactions, by signaling linear humanlikeness with a negative coding scheme up to the human–nonhuman threshold encoded in DMPFC. These activities are consistent with FFG responses to nonliving compared with living entities (e.g., tools vs animals) (Chao et al., 1999; Noppeney et al., 2006; Mahon et al., 2007; Chaminade et al., 2010). Our data suggest that preferential FFG responses to nonliving entities can be elicited gradually by different types of artificial agents. The FFG's selective humanlikeness signal was not directly expressed in psychophysical ratings but could reflect a potential input signal for neural UV computations.

Together, these data identify an apparent progression of activity patterns that reflected the transition from a linear humanlikeness continuum in TPJ, to nonlinear humanlikeness signals in DMPFC and FFG and toward a nonlinear UV value function in VMPFC. The observed functional connectivities between these areas supported this information-processing sequence. Notably, these neural signals were recorded during UV-relevant evaluations and explained interindividual variation in UV reactions.

Such linear–nonlinear transformations are known from hierarchical sensory systems that gradually, over a series of neural representations, produce selective responses to specific feature combinations (Rolls and Treves, 1998; Olshausen and Field, 2004). We found that a multiplicative combination of linear and nonlinear humanlikeness signals from TPJ and FFG was sufficient to approximate the observed neural UV representation in VMPFC. Multiplicative signal integration is biologically plausible and prevalent in sensory systems, where it produces nonlinear enhancement to specific feature combinations (Peña and Konishi, 2001; de Araujo et al., 2003; Small et al., 2004; Stein and Stanford, 2008). Based on these data, we suggest that the UV reaction can be conceptualized as a nonlinear neural valuation response elicited by a specific feature combination—high humanlikeness in nonhuman agents. This response likely derives from multiplicative integration of linear and nonlinear humanlikeness signals.

At the behavioral level, our data demonstrate how subjective UV reactions extend beyond perceptual impressions to active preference choices, guided by subjectively weighted decision attributes. To elicit UV reactions, we constructed artificial human stimuli from real human individuals that maximized humanlikeness but imposed unnatural, flawless and smoothed facial appearances and body proportions. The present neural and behavioral data suggest that this novel manipulation is particularly effective in eliciting a UV reaction, in accordance with recent suggestions (Pollick, 2010). Notably, previous behavioral studies typically reported strongest UV reactions for androids (Piwek et al., 2014; Rosenthal-von der Putten and Kramer, 2014; Rosenthal-von der Putten and Weiss, 2015; Wang et al., 2015; MacDorman and Chattopadhyay, 2016). Here, inclusion of the highly humanlike artificial humans may have elicited adaptation effects that maximized UV reactions for artificial humans while attenuating UV reactions for androids. Such adaptive coding is well established in neural valuation systems, whereby neural responses adapt to the current statistical distribution of stimuli (Tobler et al., 2005; Schultz, 2015). Future studies could test systematically how the range of presented stimuli affects UV reactions in behavior and neural activity.

Our results shed new light on the functions of different parts of medial prefrontal cortex that have been implicated in social and evaluative functions (Amodio and Frith, 2006). Previous studies reported involvement of DMPFC in evaluating traits of other people (Schiller et al., 2009; Denny et al., 2012), modeling others' values and choices (Nicolle et al., 2012; Suzuki et al., 2012; Stanley, 2016), and understanding others' intentions (Amodio and Frith, 2006; Mitchell et al., 2006). Despite these advances, the role of DMPFC in social cognition has remained elusive. Here we showed that DMPFC encoded a nonlinear humanlikeness signal that emphasized the distinction between human and nonhuman agents. This signal in DMPFC likely derived from TPJ's linear representation of the humanlikeness continuum and may have contributed to the nonlinear valuation function seen in VMPFC, as suggested by these areas' functional connections. VMPFC activity is consistently observed during value-based choice (Chib et al., 2009; Hare et al., 2010; Levy et al., 2010; Wunderlich et al., 2010; Grabenhorst and Rolls, 2011; Hunt et al., 2012; Bartra et al., 2013; De Martino et al., 2013; Zangemeister et al., 2016), including social choice (Hampton et al., 2008; Behrens et al., 2009; Suzuki et al., 2012; Sul et al., 2015). Our results advance understanding of VMPFC's functions by demonstrating how a nonlinear value function in VMPFC can be generated through multiplicative signal integration from other areas.

The amygdala is implicated in two functional domains that intersected in our study: the valuation of sensory events and the processing of social information (Phelps and LeDoux, 2005; Adolphs, 2010; Rolls, 2014). Specifically, previous studies showed amygdala involvement in face processing (Adolphs, 2010), trustworthiness evaluation (Winston et al., 2002), anthropomorphizing (Heberlein and Adolphs, 2004), and social impression formation (Schiller et al., 2009), which likely contributed to the observed amygdala activation in our decision task. Similar to VMPFC, amygdala encoding of subjects' decision variable is consistent with recent evidence implicating the amygdala in value-guided decisions (Seymour and Dolan, 2008; Grabenhorst et al., 2012; Grabenhorst et al., 2013; Hernádi et al., 2015; Grabenhorst et al., 2016). Unlike VMPFC, amygdala coding of this decision variable reflected subjects' tendencies to reject gifts from artificial agents in the UV. Thus, the amygdala may distinctly contribute to inhibiting interactions with humanlike artificial agents.

Our data suggest a novel, neurobiological conceptualization of human responses toward artificial social partners. In two experimental tasks, the VMPFC encoded an explicit representation of subjects' UV reactions as a nonlinear valuation function, by signaling selective low likability for the most humanlike artificial agents. This neural UV representation seemed to derive from a multiplicative combination of linear and nonlinear humanlikeness signals in functionally connected TPJ, FFG, and DMPFC. Thus, human reactions toward artificial agents involve a selective, nonlinear neural valuation in response to a specific feature combination (humanlikeness in nonhuman agents). These findings indicate that a basic sensory coding principle, enhanced neural feature selectivity through linear–nonlinear transformation, may also apply to human valuations of social partners, as shown here for specific artificial agents.
